# Role of Heparanase on Hepatic Uptake of Intestinal Derived Lipoprotein and Fatty Streak Formation in Mice

**DOI:** 10.1371/journal.pone.0018370

**Published:** 2011-04-04

**Authors:** David Planer, Shulamit Metzger, Eyal Zcharia, Isaiah D. Wexler, Israel Vlodavsky, Tova Chajek-Shaul

**Affiliations:** 1 Department of Medicine, Hadassah-Hebrew University Medical Center, Mount Scopus, Jerusalem, Israel; 2 Sharett Institute of Oncology, Hadassah-Hebrew University Medical Center, Ein Kerem, Jerusalem, Israel; 3 Department of Pediatrics, Hadassah-Hebrew University Medical Center, Mount Scopus, Jerusalem, Israel; Agency for Science, Technology and Research (A*STAR), Singapore

## Abstract

**Background:**

Heparanase modulates the level of heparan sulfate proteoglycans (HSPGs) which have an important role in multiple cellular processes. Recent studies indicate that HSPGs have an important function in hepatic lipoprotein handling and processes involving removal of lipoprotein particles.

**Principal Findings:**

To determine the effects of decreased HSPGs chain length on lipoprotein metabolism and atherosclerosis, transgenic mice over-expressing the human heparanase gene were studied.

Hepatic lipid uptake in hpa-Tg mice were evaluated by giving transgenic mice oral fat loads and labeled retinol. Sections of aorta from mice over-expressing heparanase (hpa-Tg) and controls (C57/BL6) fed an atherogenic diet were examined for evidence of atherosclerosis. Heparanase over-expression results in reduced hepatic clearance of postprandial lipoproteins and higher levels of fasting and postprandial serum triglycerides. Heparanase over-expression also induces formation of fatty streaks in the aorta. The mean lesion cross-sectional area in heparanase over-expressing mice was almost 6 times higher when compared to control mice (23,984 µm^2^±5,922 vs. 4,189 µm^2^±1,130, p<0.001).

**Conclusions:**

Over-expression of heparanase demonstrates the importance of HSPGs for the uptake of intestinal derived lipoproteins and its role in the formation of fatty streaks.

## Introduction

Heparan sulfate proteoglycans (HSPGs) are major components of the extracellular matrix and have a major role in signal transduction and modulating cell interactions with the microenvironment [1], [2]. Recent studies indicate that HSPGs have an important function in hepatic lipoprotein handling and processes involving removal of lipoprotein particles[3], [4], [5].

The involvement of HSPGs in multiple physiologic processes has spurred interest in identifying factors that regulate cell surface heparan sulfate (HS) content. A key regulatory enzyme is heparanase, a HS degrading endoglycosidase produced by different cell types [6], [7]. The function of the HSPG-heparanase system in modulating hepatic lipoprotein handling especially in the post prandial state has been previously studied. In the liver, infusion of heparinase (the bacterial equivalent of heparanase) into the portal vein lowers hepatic HSPG with an attendant reduction in triglyceride rich particle (TRP) clearance [8]. In diabetics, there is a reduced level of the HSPG assembly enzyme *N*-deacetylase/*N*-sulfotransferase-1 (Ndst1) that is associated with decreased post-prandial clearance of triglyceride rich particles (TRPs) and hypertriglyceridemia [9]. Based on experiments with syndecan-1 deficient mice that exhibited delayed clearance of TRLs (Triglyceride Rich Lipoproteins), Stanford et al concluded that syndecan-1 is the primary hepatic proteoglycan receptor mediating TRL clearance [10].

To study the effect of heparanase on lipid metabolism and atherosclerosis development *in vivo*, an experimental model in which heparanase is over-expressed was utilized. We have found that over-expression of heparanase induces inhibition of post-prandial lipoprotein clearance and uptake by the liver and is associated with formation of fatty streaks.

## Materials and Methods

### Animals

The study was conducted according to protocols approved by the Animal Care Committee at the Hadassah - Hebrew University Medical Center. Homozygous transgenic mice over-expressing the human heparanase gene (hpa-Tg) were produced as previously described [11]. The transgenic mice were backcrossed to C57BL/6 mice for 7 generations and maintained as homozygotes for the transgene. C57BL/6 mice were used as controls. Mice were maintained in a SPF animal facility and fed *ad libitum* either with normal chow or an atherogenic diet (42% of calories from fat, 43% from carbohydrates, and 15% from protein, TD 88137, Harlan Teklad) for six months. Food consumption was measured and there were no changes in eating behavior and body weight between pure bred hpa-Tg and control C57BL/6 mice.

### Genotyping of transgenic mice

To ensure that the animals were homozygous for the human heparanase gene, DNA was isolated from the tail tip [11] and real time PCR was performed using human hpa specific PCR primers: F-5′-TACCTTCATTGCAACACTG-3′ and R-5′-GTGACATTATGGAGGTT-3′. The PCR was performed using Syber universal PCR Master Mix (ABI, Warington, UK). L19 cDNA was used as an internal standard.

### Assessment of atherosclerosis in the aortic root

hpa-Tg and control C57BL/6 male mice were analyzed for atherosclerosis. Following perfusion of the heart, the upper section of the heart was placed in embedding medium (Tissue-Tek, Satura FineTek, Torrance, CA), frozen in methyl butane and stored at −80°C. Every other section (10 microns thick) throughout the aortic sinus (400 µm) was taken for atherosclerosis analysis. Frozen sections were stained with Oil red O and three representative sections from each mouse were analyzed by image analysis using ImagePro Plus software [12].

### Analytic procedures

Following an overnight fast, retro-orbital blood samples were taken from mice for lipid profile evaluation [13]. Cholesterol and triglyceride distribution among lipoproteins fractions was determined by fast pressure liquid chromatography (FPLC) after loading 200 µl of pooled plasma from control and hpa-Tg fasting mice onto a superose 6HR 10/30 FPLC column (Piscataway, NJ) and 0.5 ml fractions were eluted with 0.15 M NaCl, 0.01 M Na_2_HPO_4_, 0.1 mM EDTA and 0.02 mM NaN_3_. Cholesterol and triglycerides (TG) were determined using commercial kits (Roche/Hitachi, Mannheim, Germany and Thermo, Waltham, MA, respectively).

### Lipoprotein lipase (LPL) activity

Mice were injected i.p. with 0.5 U/gr heparin. Post heparin plasma was withdrawn 10 minutes later, and LPL activity was determined as described [14]. LPL activitiy is given in mU/ml, where 1 mU represents 1 nmol of free fatty acid released from the triglyceride substrate per minute.

### Fat and retinol load

An oral fat load test was performed with hpa-Tg and their controls. Mice were fasted overnight and then administered a bolus of 100 µl corn oil via a stomach tube. Triglyceride and cholesterol levels were determined in the plasma at specific time points as indicated in the text [15]. The same experiment was repeated with 100 µl corn oil containing 10 µCi of ^3^H-retinol ([11,12-^3^H(N)], Perkin Elmer, Boston) and 3000 units of unlabeled retinol (Sigma-Aldrich) [16]. Radioactivity in the plasma was determined 1.5, 3 and 6 h following fat administration. Radioactivity distribution among lipoproteins fractions was determined by fast pressure liquid chromatography (FPLC) loading a superpose column (described above) with 200 µl of radiolabeled plasma from control and hpa-Tg mice 3 h after retinol administration.

### Body distribution of radioactive retinol

Three hours after intragastric administration of bolus of 100 µl corn oil containing 10 µCi of ^3^H-retinol and 3000 units of unlabeled retinol, mice were anesthetized, bled, and perfused with a normal saline solution. Fat extraction from liver and carcass samples was done with a solution of methanol:chloroform 1:1, and radioactivity was measured in these samples and serum. Absorption of retinol from the intestine and its clearance from the circulation by liver and carcass were calculated.

### Statistical analysis

Data is reported as mean ± SEM. Comparisons between groups were made utilizing the Student's *t* test (two-tailed).

## Results

### Effect of heparanase over-expression on serum lipids

**Figure 1:**
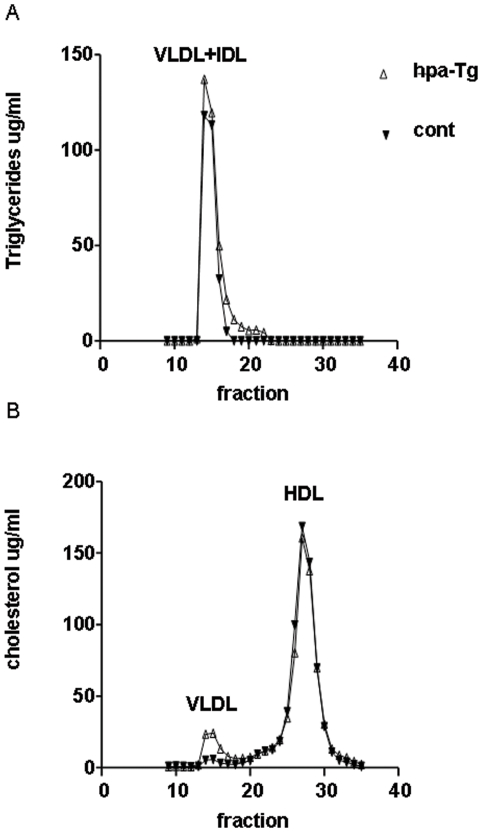
Distribution of triglyceride (A) and cholesterol (B) in the plasma of hpa-Tg (open triangles) and control C67BL/6 mice (closed triangles). Pooled plasma samples of 4 fasting mice from each group were applied to FPLC analysis. As indicated in the figure, triglyceride levels are increased in the non-HDL fractions (A), and cholesterol level is slightly increased in the VLDL+IDL fractions (B) of the hpa-Tg mice compared to controls. AUC (µg/ml x fraction) of C57BL/6 triglyceride was 281 compared to 393 of hpa-Tg whereas the AUC of C57BL/6 total cholesterol was 662 compared to 705 of hpa-Tg mice. The AUC of VLDL-cholesterol peak was 20 compared to 74 of hpa-Tg mice.

To determine the metabolic effects of increased heparanase expression, the lipoprotein profile in hpa-Tg mice fed a high fat diet was investigated. No significant difference was detected in total serum cholesterol levels between overnight fasted hpa-Tg mice (223 mg/dl ±30) and C57BL/6 mice (177 mg/dl ±18) although cholesterol levels in the transgenic mice tended to be higher (Table 1). However, there was a mild, but significant, elevation in TG levels in hpa-Tg mice compared to control mice (102 mg/dl ±11 vs. 75 mg/dl ±6, respectively; p = 0.04) as shown in Table 1. FPLC analysis revealed that the AUC of TG in the transgenic mice was increased by 1.4 fold compared to control C57BL/6 mice. A 3.7 fold increased in AUC of VLDL cholesterol was observed in the hpa-Tg mice. As most of the plasma cholesterol in mice is located in the HDL fraction, the AUC of total cholesterol didn't vary significantly between the two groups. (Figure 1A, B). There was no significant difference in post heparin plasma lipoprotein lipase activity between control and hpa-Tg mice, (40±3 mU/ml in control vs. 54±9 mU/ml in hpa-Tg mice) indicating that the effect of heparanase over-expression on remnant lipoprotein metabolism is not due to reduced lipoprotein lipase activity.

**Table 1:** Serum lipid profile of hpa-Tg and control C57BL/6 mice.

	hpa-Tg (n = 15)	C57BL/6 (n = 15)	P value
**Total cholesterol (mean±SE) mg/dl**	223±30	177±18	ns
**Triglycerides (mean±SE) mg/dl**	102±11*	75±6	0.04

### Effect of heparanase over-expression on lipoprotein metabolism

**Figure 2:**
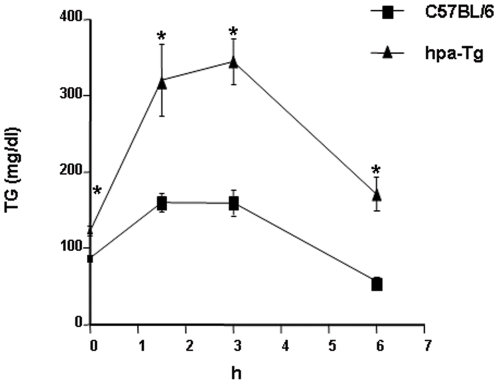
TG accumulation in plasma following oral administration. Mice were fasted overnight and then administered a bolus of 100 ul corn oil via a stomach tube. TG levels in serum of hpa-Tg mice (n = 6) were significantly higher compared to TG levels in C57BL/6 mice (n = 6) during the first 3 hrs (p<0.005 for all measured time points).

**Figure 3:**
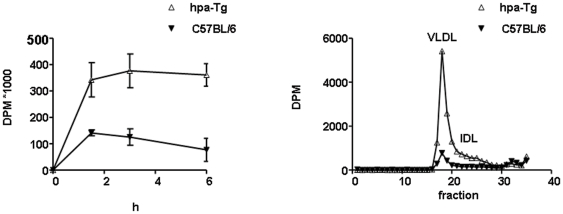
^3^H-retinol accumulation (A) and distribution (B) in plasma following oral administration. (A) Mice were fasted overnight and then administered a bolus of 100 µl corn oil containing ^3^H-retinol via a stomach tube. Radioactivity in plasma of hpa-Tg mice (n = 3) was significantly higher compared to C57BL/6 (n = 3) during the 6 h following retinol administration (p<0.01). (B) 200 µl of plasma from hpa-Tg and C57BL/6 mice, taken 3 h following retinol load were analyzed by FPLC. As indicated in the figure, retinol radioactivity was increased in the VLDL and IDL fractions of hpa-Tg compared to control mice. AUC (dpm x fraction) of hpa-Tg was 16703 compared to 4640 in C57BL/6 mice.

The effect of elevated heparanase on post-prandial lipoprotein accumulation in the plasma was studied by oral administration of corn oil to fasted mice. As shown in Figure 2A, the differences in serum TG between hpa-Tg and control mice increased during the first 3 h following the fat load. TG levels were significantly higher at all time points in the hpa-Tg mice (Figure 2A; p<0.005 for all points), and these differences were maintained up until 6 h after the fat load. For example, TG levels increased from 120 mg/dl ±10 at baseline to 320 mg/dl ±50 at 1.5 h after fat administration. This is in comparison to control C57BL/6 mice in which there was only a mild elevation in plasma TG levels measured 1.5 h post-prandially (from 90 mg/dl ±4 to 160 mg/dl ±10). We have also determined plasma radioactivity 1.5, 3 and 6 h following retinol load. Plasma vitamin A (^3^H-retinol) radioactivity was higher at all time points in the hpa-Tg compared to C57BL/6 mice (Figure 3A; p<0.01 for all points). FPLC analysis of plasma taken 3 h after retinol load revealed higher peaks of labled VLDL and remnant particles in the hpa-Tg mice (Figure 3B).

**Figure 4:**
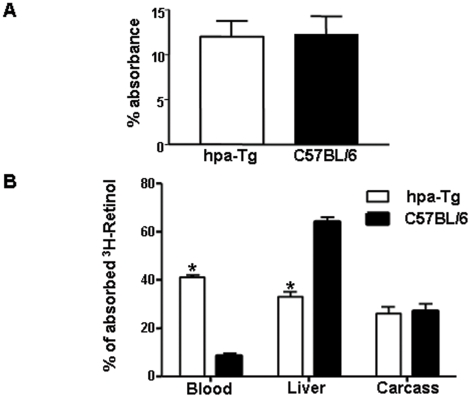
Post-prandial lipid handling in hpa-Tg and C57BL/6 mice. (A) Absorption of radio-labeled vitamin A (^3^H-retinol). Intestinal absorption was 12% of the total administered dose of ^3^H-retinol in both hpa-Tg (n = 4) and C57BL/6 mice (n = 4). (B) Distribution of absorbed vitamin A (^3^H-retinol) in blood, liver and carcass. 41% of the radioactive retinol were detected in serum of hpa-Tg mice compared to 9% detected in serum of C57BL/6 mice, p<0.0001. No difference was detected in radioactivity extracted from carcasses of the two experimental groups. The decrease in liver uptake of retinol was paralleled by an equivalent increase in the level of labeled retinol in the blood of hpa-Tg mice.

The accumulation of TG in plasma of hpa-Tg mice suggests that over-expression of heparanase decreases remnant particle clearance by the liver. To test this hypothesis, ^3^H-retinol was administered orally and the distribution of retinol was assayed in blood, liver and carcass of hpa-Tg and C567BL/6 mice. The intestinal absorption of labeled retinol was calculated by adding the radioactivity recovered in the plasma, liver and carcass (excluding other organs and the entire GI tract) and expressed as the percent of the total administered dose of radioactivity. Extraction of the administered label from liver, blood and carcass, 3 h following retinol load, revealed that the absorption was approximately 12% of the total administered dose for both the control and hpa-Tg animals (Figure 4A). However, there were differences in blood and liver distribution of labeled retinol between the two groups indicating that remnant particle clearance by the liver was reduced in hpa-Tg mice (Figure 4B). Fat extracted from liver of hpa-Tg mice contained 32% ±2 of the absorbed retinol, while fat extracted from liver of C57BL/6 mice contained 64%±2 of the absorbed radioactivity (p<0.0001, Figure 4B). The decrease in liver uptake of retinol was paralleled by an equivalent increase in the level of labeled retinol in blood of the hpa-Tg mice. The percent of the absorbed retinol found in blood was 41%±1 in hpa-Tg mice compared to 9%±1 in controls (p<0.0001).

### Fatty streaks formation in heparanase over-expressing mice

**Figure 5:**
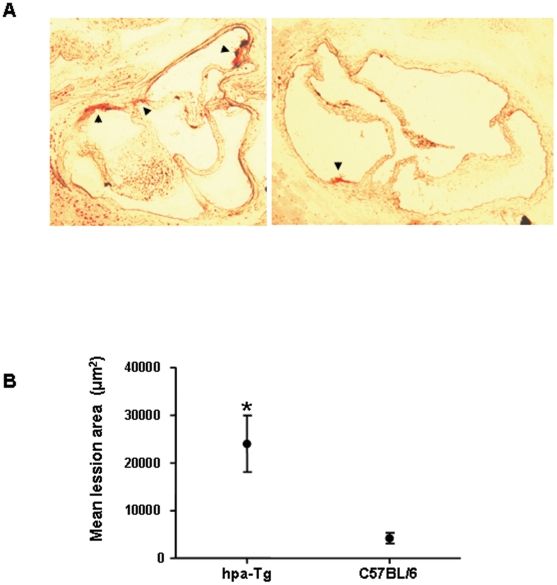
Fatty streaks in hpa-Tg and C57BL/6 mice. (A) Frozen sections of aortic root from hpa-Tg and C57BL control mice (n = 15 for each group) were stained with Oil-Red-O for lipid content. (B) Mean lesion area in heparanase over-expressing mice was significantly higher compared to control mice, p<0.001.

The effect of over-expression of heparanase on the development of atherosclerotic lesions was studied in hpa-Tg and control C57BL/6 male mice fed a high fat diet for 6 months. Frozen sections of aortic root were stained with Oil-Red-O for lipid content and fatty streak formation. Figure 5A shows representative sections of aorta from hpa-Tg and control mice. Quantitative analysis of fatty streaks was performed by measuring the extent of Oil-red-O staining of cryosections from the aortic root. Mean lesion cross-sectional area in heparanase over-expressing mice was almost 6 times higher when compared to control mice (23984 µm^2^±5922 vs. 4189 µm^2^±1130, p<0.001; Figure 5B).

## Discussion

By manipulating the expression of heparanase, the only HSPG degrading enzyme, it is possible to determine the effect of decreased intact HSPG content on both liver uptake of TRPs and the development of atherosclerotic lesions. Our studies show that when HS chain length is decreased due to over-expression of heparanase, there is increased fasting TG and reduced hepatic uptake/clearance of TRPs. At the same time, the cross sectional area of fatty streaks was increased.

HSPGs have been shown to regulate the clearance of TRPs via binding, uptake and degradation of these particles in the liver. The importance of HSPG mediated pathways of TRP clearance was demonstrated by several studies including Ebara et al [9] who demonstrated in a diabetic murine model that delayed clearance of post-prandial apoB-48–containing lipoproteins is due to decreased hepatic HS. Reduced hepatic HS in diabetes was attributed to decreased expression of hepatic N-deacetylase/N-sulfotransferase 1 (NDST) which is a key enzyme in the biosynthesis of HSPG [9], [17]. Our data demonstrates that over-expression of heparanase is associated with delayed clearance of TRPs and reduced uptake by the liver resulting in elevated plasma levels of these atherogenic particles.

The specific role of HSPGs in the hepatic uptake of lipoproteins, especially TRPs, has been elucidated by selectively modifying different receptors and enzymes involved in lipoprotein metabolism. In the most recently proposed models, apoE mediates the binding of remnants to HSPGs leading to sequestration of these particles in the space of Disse. Internalization of remnants occurs via several pathways including those transduced by LDL receptor (LDLR), LDLR-related protein (especialy in the absence of functional LDLR) and HSPG [3], [4], [18]. HSPGs also facilitate internalization of apoE-encriched remnant lipoproteins by a LDLR independent pathway [2]. At least two distinct HSPGs, syndecan and perlecan, have been shown to be involved in the internalization of remnants in tissue culture system [19], [20]. Stanford et al. provided direct genetic evidence *in vivo* that syndecan-1 is the primary hepatic proteoglycan receptor mediating TRL clearance. Their conclusion was based on the observation that TRLs resembling VLDL and VLDL remnants accumulate in fasted syndecan-1 deficient mice and that these mice exhibited delayed clearance of TRLs derived from dietary fat [10].

The significance of the contribution of HSPGs to remnant clearance was shown by Ji et al. [8]. Infusion of heparinase into the portal vein of mice reduced the level of hepatic HSPG (as measured by sulfation) leading to a significant decrease in clearance and uptake (up to 80%) of remnants. Similarly in our experiments with human heparanase over-production, there was a significant reduction in remnant clearance and internalization. Interestingly, in contrast to Ji et al., we have previously shown that overproduction of heparanase does not lead to overall decrease in hepatic HSPG content but to a reduction in the length of the HS chains attached to the proteoglycan core protein [21]. Shortening of the HS chains may have a significant impact on remnant clearance as there are fewer sites available for the binding of lipoproteins resulting in less sequestration and internalization.

LPL is known to accelerate the clearance of post-prandial lipoprotein by hydrolyzing the TG content of the TRPs. It also binds to lipoprotein *in vivo*, independent of its lipolytic activity and serves as a bridge between lipoprotein and HS chains [22]. The association between hypertriglyceridemia and atherosclerosis development has been demonstrated recently in mice that are mutated in chylomicron-processing due to mutations in LPL binding or activity and accumulation of large TG-rich particles [23], [24]. We investigated whether over-expression of heparanase impacts on the availability of LPL on the endothelial surface thereby reducing LPL mediated lipolysis of TRPs and binding to hepatic HSPGs. Our data showed that there was no difference in post heparin plasma LPL activity between hpa-Tg and control mice indicating that the effect of heparanase over-expression on remnant lipoprotein clearance is not due to reduced lipoprotein lipase activity and availability. This is consistent with recent findings that glycosylphosphatidylinositol-HDL binding protein 1 (gpihbp1) is the main protein that binds LPL to endothelial cell surface rather than HSPG as has been previously reported [5], [25], [26].

Thirty years ago, Zilversmit proposed that post-prandial TRPs promote the formation of atherosclerotic lesions [27] based on experimental data showing that cholesterol fed rabbits which develop atherosclerosis have a defect in chylomicron clearance by the liver. Subsequent human studies indicated that triglyceridemia is an independent risk factor for early atherosclerosis and subjects with coronary artery diseases exhibit delayed metabolism of TRPs [28], [29], [30]. Part of the increased susceptibility to atherosclerosis of patients with type 2 diabetes mellitus and the metabolic syndrome has been attributed to the decreased clearance of TRPs [5], [31], [32]. TRPs are extremely atherogenic and there is direct evidence showing high levels of TRPs in human atherosclerotic plaques [33], [34]. Indeed, we show that in hpa-Tg mice the increased TG levels and decreased clearance of post-prandial lipoprotein remnants by the liver are associated with increased cross sectional area of fatty streaks.

It should be noted that mice are athersoclerosis resistant species and most studies on murine models have been performed utilizing either apoE-KO or Ldl-r-KO null backgrounds. We were unsuccessful in developing hpa-Tg x apoE-KO or hpa-Tg x Ldl-r-KO lines despite trying for over a year. Notwithstanding the limitations of the dietary model of murine atherosclerosis, we were able to show the effects of heparanase over-expression on lipoprotein metabolism and fatty streak formation. From a different perspective, there are advantages to the dietary model as it is more reflective of environmental causes of atherosclerosis.

Over-expression of the heparanase gene indicates the importance of HSPGs for the uptake of TRPs and its protective effect on fatty streak formation and potentially atherosclerosis initiation. From a clinical perspective, changes in HS structure and/or expression may contribute to atherosclerotic manifestations in patients predisposed to mild but clinically relevant hypertriglyceridemia. Potential pharmacologic modulation of heparanase expression or activity may retard the development of atheromas. For example, heparanase inhibitors that are being developed as anti-cancer drugs such as PI-88 [35] and glycol-split heparins [36] may also be useful in the treatment of atherosclerosis. Further characterization of animal models in which HSPG expression and function is modulated will provide additional insights both into the mechanisms of atherosclerosis and potential intervention for treating atherosclerotic disease.
